# The Effect of Music Therapy on Psychological Outcomes for Neurological Conditions: A Systematic Review

**DOI:** 10.3390/medicina61091611

**Published:** 2025-09-05

**Authors:** Sarah H. Gardener, Elizabeta B. Mukaetova-Ladinska, Nellinne Antoinette Perera

**Affiliations:** 1 School of Psychology and Vision Sciences, University of Leicester, University Road, Leicester LE1 7RH, UK; lnar2@leicester.ac.uk; 2 The Evington Centre, Leicester General Hospital, Gwendolen Road, Leicester LE5 4QF, UK

**Keywords:** music therapy, neurological conditions, cognitive function, mood, emotion, behaviour, quality of life

## Abstract

*Background and Objectives:* Music therapy has been used as a non-pharmacological treatment for neurological conditions, supporting cognitive, psychosocial, behavioural and motor functions. Although it is evidence-based, safe, and cost-effective, music therapy remains uncommon in neurorehabilitation services, largely due to a lack of quality research. This review aims to understand the effect of music therapy on psychological outcomes for neurological conditions using randomised clinical trials (RCTs). *Materials and Methods:* A systematic review of literature published in four databases (PsycINFO, CINAHL, PubMed, and Scopus which is inclusive of Medline and EMBASE) from 1 January 2015 to 31 January 2025 was performed. *Results:* Ten RCT’s comprising 469 participants were included in this review, with seven studies synthesised using a systematic review without meta-analysis approach. MT was found to improve cognitive function in individuals with Parkinson’s disease (PD) and Traumatic Brain Injury (TBI), but not in those with stroke or Huntington’s disease (HD). In HD, improvements in cognitive function were noted in the comparator group. MT led to significant improvements in emotional well-being in stroke survivors, though not in mood. Behavioural outcomes did not significantly improve in HD, while quality of life improved following MT for individuals with multiple sclerosis (MS) and stroke. *Conclusions:* Overall, MT was perceived as a positive intervention with potential psychological benefits. However, findings were mixed, and the heterogeneity of population, intervention, comparator, outcomes, and study design limited the ability to detect group differences and reduced confidence in the findings. Future research should focus on larger-scale RCTs that reduce bias, accommodate population diversity, and clearly define and distinguish MT from treatment-as-usual. Better standardisation of outcomes and improved reporting will be essential to evaluate the efficacy of music therapy in neurological conditions and strengthen the evidence base for its use in clinical practice.

## 1. Introduction

Music therapy (MT) is characterised by the use of music within a therapeutic relationship with the music therapist, encompassing multisensory stimulation [1,2,3,4,5]. MT, delivered by qualified, trained music therapists is distinguished from “music-based interventions” delivered by healthcare professionals that often comprises music listening, where intervention content varies depending on professional background [2,6].

MT supports psychological factors associated with neurological dysfunction, such as mood, emotion and distress management, and quality of life, while facilitating empowerment and enabling a supportive role towards others, which in turn support other areas of neurorehabilitation such as motor function [7,8,9,10,11,12,13,14]. Neurologic music therapy (NMT) is a specific evidence-based form of MT that supports many presentations of a wide range of neurological conditions that affect the brain, spinal cord and nerves, such as traumatic brain injury (TBI), stroke, Parkinson’s disease (PD), and multiple sclerosis (MS), among many others, by improving disorder of consciousness awake levels, to aiding sensorimotor, speech and language, and cognitive rehabilitation [11,15,16,17,18,19,20]. Therefore, MT is unique in its versatility, and NMT can be used to support a range of needs during neurological rehabilitation [21].

### 1.1. Music Therapy in Theory and Clinical Practice

**Figure 1:**
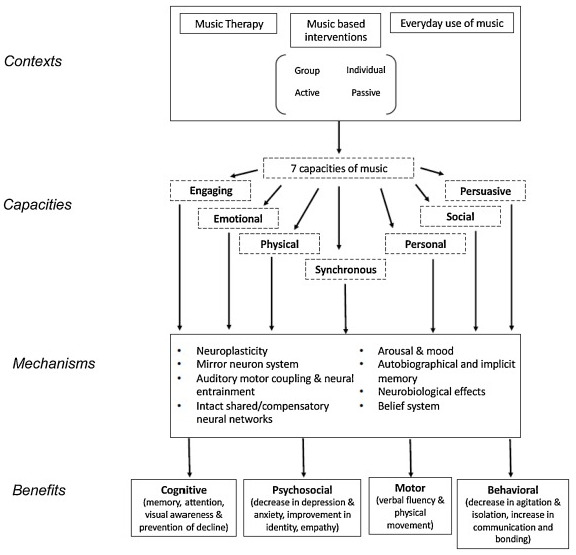
The Therapeutic Music Capacities Model (TMCM) [15]. This framework illustrated neurological and psychological mechanisms that link the capacities impacted by MT to cognitive, psychosocial, motor, and behavioural benefits.

The rational scientific mediating model (RSMM) was designed to translate music research into applied clinical music research. This led to the development of the transformational design model (TDM), used to translate clinical music research into functional MT [21,22,23]. This ensures therapeutic goals are both prospective rather than retroactively incorporated, a weakness of some music interventions, and better related to the functional therapeutic outcomes [21,23]. To further explore how MT benefits neurological conditions and establish a theory of change, the introduction of the Therapeutic Music Capacities Model (TMCM) was a step further and illustrates the neurological and psychological mechanisms that link the capacities impacted by MT to cognitive, psychosocial, motor, and behavioural benefits. In addition, this model provides a framework how MT can be personalised, adapted, and applied to all neurological conditions (Figure 1) [15,24,25].

MT is an evidence-based intervention for neurorehabilitation. However, limited resources are available for MT use in the United Kingdom (UK) National Health Service (NHS) [26]. MT research has a greater focus on dementia compared to other neurological conditions, where it is more widely used, suggestibly due to limited pharmacotherapy options for dementia unlike other neurological conditions that can be partially managed by pharmacological and multimodal non-pharmacological interventions (i.e., physiotherapy, speech and language therapy, electrotherapy etc.) [27,28,29,30] MT is not currently commissioned in specialised neurorehabilitation services for neurological conditions other than dementia, where in the latter there is still limited funding [31,32]. This raises ethical concerns about pharmacotherapy being heavily relied upon and promotes exploration of MT as an effective nonpharmacological treatment that is less invasive, side-effect-free and cost-reducing intervention in the treatment of neurological disorders [11,15].

### 1.2. Research in Music Therapy for Neurological Conditions

Neurological conditions impede certain cognitive functions (i.e., executive functioning or speed of processing) impacting mental health, with them being more disabling than physical impacts in some instances [8,13]. MT has been suggested to improve mood and motivation, potentially increasing treatment adherence, implying psychological improvements from MT may benefit other domains, such as motor function [33]. Studies on MT for psychological benefits, such as cognitive rehabilitation, are limited, though cognition, mood, emotion, and quality of life for neurological condition have all been shown to benefit [11,17,34].

For more detals, please see the main text. 

Neurobiological and clinical studies provide further evidence for MT benefits in neurological and psychiatric disorders. Thus, musical experiences are associated with sustained brain volume and activation of networks, especially those involved in executive function, memory, language processing and regions associated with reward, motivation, arousal, and emotions (i.e., frontal and hippocampal brain areas along striatum, midbrain and amygdala). This provoked engagement of different brain circuits is achieved by inducing and promoting neurogenesis and neuroplasticity, as well as inducing neuromodulators involved in pleasure (dopamine), seeking reward (dopamine and opioid system). mood, alertness, motivation (serotonin and noradrenaline) and memory (acetylcholine and serotonin) [35,36]. Via knowing the molecular changes music has upon distinct neural circuits, it may be possible to target specific NMT interventions corresponding to a patient’s individual needs (for instance strengthening neural network connections between auditory and motor or affective/motivational/sensory systems in stroke, traumatic brain disorders, multiple sclerosis, movement disorders, such as Parkinson’s disease, etc). This potential needs to be explored further in neurorehabilitation programmes in the future.

Research on MT is generally low in quality, potentially attributed to challenges with evaluating this procedure [6,37,38,39,40]. For example, the concept of MT has varying definitions across studies, and it is difficult to distinguish the MT effects from other treatment-as-usual interventions [17,39]. Outcome measures have been found to vary greatly between studies, but include the use of frequently used, standardised psychological measures with accepted psychometric properties in wider research and clinical practice such as the Mini Mental State Examination (MMSE) [34]. Even when other controlling for variation, music therapists bring their own training, musicality, style, and experience, and embrace creativity, which is an asset but difficult to quantify for research [40]. Furthermore, with few UK NHS services offering MT, research opportunities are limited [41,42]. The National Institute for Health and Care Excellence (NICE) (2023) guidelines [43] recommend research on MT should include randomised-controlled trials (RCTs) to establish benefits for neurorehabilitation and patient-centred outcomes [44]. Therefore, this review will critique RCTs exclusively.

With the literature on dementia establishing MT as a recommended nonpharmacological intervention to support psychological outcomes [45,46] it is important to review other neurological conditions, and whether a shift from potentially unnecessary pharmacotherapy is possible [2,25]. The objective of this review will establish the evidence and quality of research on MT for neurological conditions that meet criteria for UK specialised neurorehabilitation services, using the recommended RCT design [31,43]. Limitations and recommendations will be identified for future research and clinical implications. The research question is to review whether there is an effect of MT for psychological outcomes in adults with neurological conditions.

## 2. Method

### 2.1. Search Strategy

To evaluate the effect of MT for psychological outcomes in adults with neurological conditions, the PICOS framework, well-established for developing systematic research, was employed to identify search terms and inclusion and exclusion criteria, consisting of the population (P) as adults with neurological conditions, intervention (I) as MT, comparison (C) as treatment-as-usual or specific alternative intervention, clinical outcome of interest (O) as psychological factors (see Table 1), and study design (S) as RCTs [9,10,11,12,15,19,47,48,49,50,51].

The search strategy used search terms and synonyms of key terms including music therapy and types of neurological conditions (Tables S1 and S2) in representative databases frequently used in MT research: PsycINFO, CINAHL, PubMed, and Scopus (inclusive of Medline and EMBASE), covering psychology, healthcare, and peer-reviewed research [39,58]. Non-peer-reviewed research, including grey literature, was excluded due to risk of poor validity and unoriginality of research [59,60].

Five specific inclusion and exclusion criteria were used to determine eligible studies for inclusion in the review (Table 2). All neurological conditions were included in the search, except for dementia, spinal cord injuries, and neurodevelopmental conditions. Search results were filtered between 1 January 2015 and 31 January 2025 to obtain the most recent research in the field worldwide. This captures a time when MT is a registered profession requiring formal training, and before, during and after the COVID-19 pandemic when alternative forms of delivering MT were required, such as distance, instrument-use limitations, and online delivery, which remain incorporated clinically and therefore relevant to current practice [7,32,61,62,63]. Limiting the search to English language benefits time constraints, reduces heterogeneity, and is the common language used in MT reviews, although may bias the range of studies despite opening the search to any location [64,65,66]. RCT-only studies were included since RCTs are recommended to improve the quality of findings establishing the effects of MT for neurological conditions [35,36,43]. In the current review, RCT pilot studies were included with data relevant to MT effectiveness [67]. Furthermore, RCT pilot studies are increasingly comparable to main studies [68]. Five of the ten papers [11,33,53,54,55] included outcome measures not directly relevant to the research question but were included to ensure relevant data were captured and because different outcomes can be related since MT may be integrated into a multi-disciplinary approach [42]. However, the combined outcomes in these studies were accounted for. MT for dementia, including Alzheimer’s disease, dementia with Lewy body, vascular dementia, mixed dementia, etc., and neurodevelopmental conditions [i.e., autism spectrum disorder, attention-deficit/hyperactivity disorder (ADHD), intellectual disability, specific learning and tic disorders] are better established and follow differing treatment pathways to other neurological conditions, and were therefore excluded [29,31,69]. Spinal cord injuries have been excluded to focus on neurological conditions where there are direct deficits on psychological factors such as cognition [70]. Since RCTs require quantitative data for increased comparability and generalisability, qualitative studies were excluded [71]. Reviews were excluded due to the research not being original, and protocols were excluded due to lack of data. Feasibility studies have been excluded with aims observing intervention feasibility rather than effectiveness. Studies researching non-psychological outcomes such as motor or physical were also excluded.

### 2.2. Search Terms

To ensure interventions were conducted by music therapists, the search terms were limited to “music* therap*” rather than “music” or “music-based interventions” (Table S1). This accounted for any relevant specific forms of MT, such as NMT [5]. Robb et al. (2018) [6] explain the challenges in comparing music intervention research due to poor reporting of interventions; therefore, it is important to ensure interventions are evidence-based. Consequently, only evidence-based MT interventions delivered by trained music therapists or professionals qualified to practice NMT were included.

**Figure 2:**
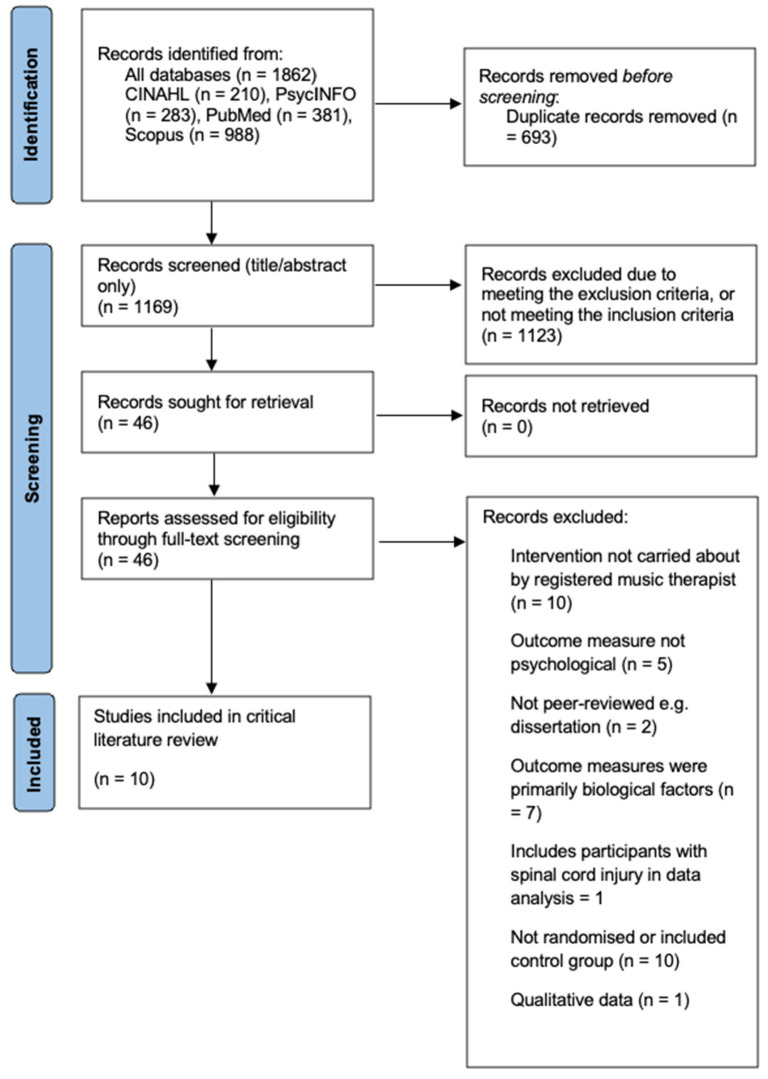
PRISMA flowchart demonstrating the process of paper identification for review [72].

Duplicates were removed manually using the Endnote function. Relevant data were extracted, and 10 papers included, illustrated in the Preferred Reporting Items for Systematic Reviews and Meta-analyses (PRISMA) flowchart (Figure 2) [72]. The small number of papers may reflect a lack of RCTs conducted on MT, hence the recommendations for research in the NICE guidelines [43]. Abstracts of identified articles and full texts of studies were independently screened and reviewed by two reviewers (SG, NAP) with disagreements resolved by discussion.

### 2.3. Data Synthesis and Quality Appraisal

The Revised Cochrane Risk-of-Bias version 2 Tool for Randomised Trials, recommended and designed for assessing risk of bias in RCTs [73,74,75] was used for quality appraisal. The Synthesis Without Meta-analysis (SWiM) reporting guidelines were applied to the synthesis of data to answer the review aim, accounting for limited reporting of results [24]. The standardised metric was vote count for direction of effect, tabulated and determined by *p*-value significance and direction [24]. Combining *p*-values was not possible as a precise *p-*value for each outcome was not consistently available [24] Average intervention effect sizes in a meta-analysis was not possible without sufficient data [76]. Data extracted included study characteristics (sample size, gender, age, location, outcome measures, type of MT) (Table 3), followed by data synthesis, where possible extracting the direction of effect, *p*-values, confidence intervals, and calculating Cohen’s *d* effect size using mean scores, standard deviation, and number of participants [24,77] (Table S2).

### 2.4. SWiM Approach

The characteristics table, with informal heterogeneity investigation, and data synthesis tables helped grouped neurological conditions to show variation across conditions [48,53] (Table 3, Tables S1–S3). The effect direction table also grouped studies by psychological outcome (Table S3). Studies were further excluded in the synthesis if between-group analyses were not undertaken since the intervention could not be reliably compared with the comparator [78,79].

To assess certainty of findings, the Gradings of Recommendations Assessment, Development, and Evaluation (GRADE) principles were applied narratively [76,80,81]. Limitations of the vote count direction of effect did not provide data for analysis of GRADE domains such as consistency, therefore a rank was not applied [76]. Furthermore, with a small number of studies and variety of psychological outcome measures, quantifying proportion of effects and indicating sample size visually has limited value and risks deception.

The systematic review is compliant with the PRISMA guidelines (Figure 2, Table S4). The study was not registered on the PROSPERO resource and has not a prepublished protocol.

## 3. Results

### 3.1. Characteristics of the Included Studies

Ten studies were included in the review and their characteristics are displayed in Table 3. The sample sizes ranged from 27 to 82 participants, with an averaging age of 41–73 years. Each study recruited one neurological condition, including stroke, multiple sclerosis (MS), Parkinson’s disease (PD), traumatic brain injury (TBI), and Huntington’s disease (HD). The treatment consisted of specific types of MT or NMT, delivered over 1–24 sessions. The MT and NMT interventions were delivered by trained music therapists, except for studies by Impellizzeri et al. [52,82], where interventions were delivered by NMT trained neuropsychologists. Comparators were treatment-as-usual (standard multi-disciplinary neurorehabilitation), except for four studies comparing MT to an alternative intervention [33,54,55,56].

The psychological outcomes were assessed using different self-report and observational standardised measures, at baseline and after intervention in all studies, with three studies evaluating follow-up outcomes at 3–6-month time points [33,53,54] (Table 3).

Outcome measures: Beck Depression Inventory-II (BDI-II), Mini-Mental State Examination (MMSE), Modified Rankin Scale (MRS), Barthel Index (BI), Trail-making test part B (TMT-B), Forward digit-span test (FDST), General self-efficacy scale (GSES), Multiple affect adjective check list revised (MAACL-R), Self-assessment manikin (SAM), Brief repeatable battery of neuropsychological test (BRB-N), Multiple sclerosis quality of life-54 (MSQOL-54), Beck depression inventory (BDI), Emotion awareness questionnaire (EAQ), McClelland motivational factors (MMF), Montral Cognitive Assessment (MoCA), Hamilton Rating Scale for Depression (HRSD), Frontal Assessment Batter (FAB), Hoehn and Yar Stage Scale (HY), Geriatric Depression Scale (GDS), Voice-Related Quality of Life (VRQOL), Visual Analogue Scale for Mood (VASM), Medical outcome study 36-item short-form health survey (SF-36), Stroke-adapted 30-item version of the sickness impact profile (SA-SIP30), Hospital Anxiety and Depression Scale (HADS), Italian version of McGill Quality-of-Life Questionnaire (MQOL-It), Behaviour Rating Inventory of Executive Function (BRIEF), Sustained Attention Response Task (SART), Wechsler Memory Scale-Revised (WMS-R), Rey Auditory Verbal Learning Test (AVLT), Apathy Evaluation Scale (AES), Profile of Mood States (POMS), Stroke Impact Scale (SIS), Treatment Self-Regulation Questionnaire (TSRQ), Intrinsic Motivation Inventory (IMI), Sustained Attention to Response Task (SART), Wechsler Adult Intelligence Scale IV (WAIS-IV), Wechsler Memory Scale III (WMS-III), Behaviour Observation Scale Huntington (BOSH), and Problem Behaviours Assessment-short version (PBA).

### 3.2. Quality Appraisal and Risk of Bias

The Cochrane Risk of Bias (RoB) 2 tool [75] was used to evaluate the quality of the included studies (Table S3).

### 3.3. Randomisation

All participants were randomly allocated into intervention and comparator groups, using computerised randomisation methods to minimise bias. Despite this, two studies [11,52] did not detail the randomisation process or whether allocation was concealed, but deemed low risk of bias with no baseline group differences [75,83].

Three studies indicated a single difference between intervention and comparator groups at baseline [52,53,56]. Thus, Van Bruggen-Rufi et al. (2017) [56] found one significant difference for Total Functional Capacity [this classification separates Huntington disease stage (as an inclusion criterion or endpoint in clinical trials accepted by the Food and Drug Administration], but no difference when adjusting means, suggesting it did not confound results. Siponkoski et al. (2020) [53] reported the cause of injury differed between groups but this item was not clinically important. These single baseline differences are therefore likely attributed to chance [52,53,75].

### 3.4. Deviations from Intended Interventions

Concealing group allocation from participants and intervention deliverers was not possible due to the nature of MT. Inpatient settings are relatively controlled environments compared to community, and although it may be easier to monitor deviations from intended interventions, control group participants are more likely to be exposed to the intervention accidentally, increasing risk of bias [58] (Table S5).

MT interventions were adapted to participant-centred needs, adjusting difficulty levels and intensity [33,53]. This would be expected in clinical practice and therefore was not deemed biased, and not adapting to needs could bias a negative effect. Adapting to participant needs without biasing intervention relies on therapist experience, hence the importance for trained music therapists to deliver MT.

**Table 3:** Characteristics of the included studies.

	Study Design	Sample Size	Age (Mean Years)	Sex (Female)	Neurological Condition	Location and Research Setting	Outcome Measures	Music Therapy Intervention	Comparator
Chou et al. (2024) [84]	RCT pilot study	82	58	28%	Stroke	Taiwan, inpatient setting	BDI-II, MMSE, MRS, BI Timepoints: Before and after intervention	Neurologic Music Therapy—Therapeutic Singing, Melodic Intonation Therapy, Rhythmic Speech Cueing, Therapeutic Instrument Music Playing, Music Cognitive Training (from neurologic music therapy) (in addition to treatment as usual) Frequency: Four hours over four weeks (extra to neurorehabilitation as normal)	Conventional therapy (treatment as usual)
Haire et al. (2021 [55]	RCT	30	55.9	47%	Stroke	Toronto, Canada, community setting	TMT-B, FDST, GSES, MAAC-R, SAM Timepoints: Two baseline assessments one-week apart. One post-intervention assessment.	Therapeutic Instrumental Music Performance (TIMP) Frequency: Three times a week for three weeks	The comparator groups consisted of TIMP plus cued motor imagery and TIMP plus motor imagery without external cues
Poćwierz-Marciniak & Bidzan (2017) [57]	RCT	61	64	78.7%	Stroke	Gdynia, Poland, inpatient neurological rehabilitation hospital	SF-36, SA-SIP30, Cantril Ladder Timepoints: Before and after intervention	Cognitive Music Therapy, Guided Imagery and Music, 1:1 Frequency: Twice a week for five weeks	Standard care (physiotherapy, ergotherapy, psychological diagnosis, maintenance psychotherapy)
Raglio et al. (2017) [11]	RCT pilot	38	72.7	58%	Stroke	Pavia, Italy, inpatient neurological rehabilitation hospital	HADS, MQOL-It Timepoints: Before and after intervention	Relational Active Music Therapy (RAMT) Frequency: Three sessions per week, 20 sessions total	Standard care (physiotherapy, occupational therapy)
Segura et al. (2024) [33]	RCT	58	63.2	24%	Stroke	Barcelona, Spain, ex-inpatient neuro-rehabilitation	BRIEF, SART, Figural Memory subtest from the WMS-R, AVLT, Verbal Fluency test in Spanish, BDI-II, self- and informant-version of AES, POMS, SIS, TSRQ, IMI, Strategies Used to Promote Health Timepoints: Before and after intervention, with 3-month follow-up	Enriched Music-supported Therapy Frequency: Once a week music therapy, plus three weekly individual self-training session, for 10 weeks	Graded Repetitive Arm Supplementary Program (GRASP) only
Impellizzeri et al. (2020) [82]	RCT pilot study	30	51	37%	Multiple Sclerosis	Messina, Italy, clinic centre setting	BRB-N, MSQOL-54, BDI, EAQ, MMF Timepoints: Before and after intervention	Neurologic Music Therapy—Associative mood and memory training, Music in psychosocial training and counselling (half of the treatment-as-usual time replaced with music therapy intervention) Frequency: Three times per week for 8 weeks	Treatment-as-usual (same number of sessions as the music therapy group)
Impellizzeri et al. (2024) [52]	Pilot Quasi-RCT	40	62.45	30%	Parkinson’s disease	Messina, Italy, clinic centre setting	MoCA, HRSD, FAB, Stroop test, Visual search test Timepoints: Before and after intervention	Computer-Assisted Rehabilitation Environment (CAREN), Rhythmic Auditory Stimulation, Therapeutic Instrumental Music Performance Frequency: Three sessions per week for 8 weeks	Standard treatment with CAREN selected scenarios three times per week
Lee et al. (2024) [54]	RCT	27	73.3	52%	Parkinson’s disease	Arizona, USA, Tremble Clefs therapeutic singing group	HY, GDS, VRQOL, VASM Timepoints: Before and after intervention (VASM only)	Therapeutic Group Singing (TGS), Straw Phonation Combined with Therapeutic Singing (SP + TGS) Frequency: Single session	Speaking-only control group
Siponkoski et al. (2020) [53]	Cross-over RCT	40	41.3	41%	Traumatic Brain Injury	Helsinki, Finland, brain injury clinic setting	FAB, Number-Letter Task, Auditory N-back Task, Simon Task, SART, Similarities, Block Design, and Digit Span subtests of the WAIS-IV, Words Lists I and II subtests of the WMS-III Timepoints: Before and after intervention, follow-up (3 and 6 months)	Rhythmical Training, Structured Cognitive-motor Training, Assisted music playing Frequency: Twice per week, for 20 sessions	Standard care (physiotherapy, occupational therapy, neuropsychological rehabilitation, speech therapy)
Van Bruggen-Rufi et al. (2017) [56]	RCT	63	54.4	68.3%	Huntington’s disease	Netherlands, set in four specialised Huntington’s disease care facilities	BOSH—social-cognitive functioning subscale and the mental rigidity and aggression subscale, PBA Timepoints: Before intervention, halfway (8th session), end of intervention (16th session), follow-up (12 weeks after intervention)	Followed protocol “music therapy for Huntington’s patients on improving and stimulating communication and self-expression” Frequency: One session per week, for 16 weeks	Recreational therapy (with treatment guide offered in same circumstances as music therapy group e.g., reading the newspaper, cooking, arts and crafts, handwork, puzzles/games)

In Chou et al. (2024) [84], one (therapeutic instrument music playing) of five (therapeutic singing, melodic intonation therapy, therapeutic instrument music playing, rhythmic speech cueing and music cognitive training according to the patient’s abilities) MT interventions were not completed for an unknown number of participants due to time constraints, potentially impacting magnitude and direction of effect. Similarly, Haire et al. (2021) [55] carried out nine of the twelve planned intervention sessions, but this was changed prior to starting the intervention without deviation, unlike Chou et al. (2024) [84].

Three studies saw withdrawals, and intention-to-treat (ITT) analyses were used to minimise bias [33,53,56]. Siponkoski et al. (2020) [53] used multiple imputation, considered a reliable method with missing data [85]. Segura et al. (2024) [33] used multiple imputation with a high missing data rate, reducing reliability, but found similar results between ITT and per-protocol analyses, strengthening reliability, while reporting the ITT analysis, deemed the least biased [33,44,86].

### 3.5. Missing Outcome Data

All missing data were accounted for across studies. Siponkoski et al. (2020) [53] considered their data missing at random and used parallel datasets to minimise sampling variability [75] Despite this, in the Van Bruggen-Rufi et al. (2017) study [56], some participants withdrew due to lack of motivation. However, the authors did not state the numbers in each group, therefore it is not clear if this reason relates to MT or lack of MT. However, linear mixed model analyses are less sensitive to missing data, therefore this may have impact on the results [56,87].

### 3.6. Measurement of the Outcome

Consistent standardised measures were used to evaluate psychological outcomes before and after intervention for all participant groups across studies. However, given the range of studies used and locations included in this review, some measures have reduced validity and reliability following translation and cultural adaptations. For example, Segura et al. (2024) [33] translated the Profile of Mood States scale into Spanish, where meaning of psychological constructs may differ from the original measure in English, and therefore should be considered during interpretation [88]

Concealment of allocation was not possible for participants and therapists, raising some concerns for most studies. Bias reduced by concealing participant allocation from assessors across studies, with three exceptions. Lee et al. (2024) [54] assessors were aware of allocation and familiar with participants, increasing potential bias for participants seeking to please facilitators when assessing outcomes. Similarly, in the Van Bruggen-Rufi et al. study (2017) [56], some assessors were blinded to participant allocation but nursing staff assessing behaviour outcomes may have been aware and influenced by allocation [75]. Poćwierz-Marciniak and Bidzan (2017) [57] assessors also delivered the intervention; however, measures were self-report not observer-dependent, minimising this risk of bias. Nonetheless, allocation awareness may have influenced participant-reported outcomes, raising some concerns for all studies except Siponkoski et al. (2020) [53], where no self-report measures were conducted, and assessors were blinded (Table S5).

### 3.7. Selection of the Reported Results

Some studies bias towards reporting significant over non-significant findings [48,74]. For example, Impellizzeri et al. (2024) [52] do not discuss the non-significant Hamilton Depression Scale outcome, and only report statistically significant *p*-values without clear reporting of full statistical tests, suggesting optimism bias [89]. Similarly, several studies discuss significant outcomes where only select subtests for the outcome were significant, without balance for non-significant subtests [33,54,55,56,57,82]. This indicates bias towards rejection of the null hypothesis, increasing risk of type I error [90].

Finally, studies carrying out an ITT analysis demonstrated how planned analysis was altered to account for participant drop-outs [33,53]. Van Bruggen-Rufi et al. (2017) [56] followed the analytical procedure in the separately published protocol, but other studies did not detail planned analysis or refer to protocols [11,52,54,55,57,82,84,91]. As a result, there is potential bias towards reporting of significant findings, however, reported results were based on the planned outcomes measured, suggesting low risk of bias from changes to analysis [75].

### 3.8. Data Synthesis and Key Findings

Seven studies included in the vote count of estimated direction of effect were used to determine whether MT had an effect for each psychological outcome. Data related to the research question were extracted, excluding data irrelevant to the review although considered when interpreting author’s conclusions [11,33,53,54,55]. Effect size has been calculated for outcomes where data were provided, indicating the magnitude of effect for individual outcomes, but these were not synthesised due to lack of data across studies. If time constraints allowed authors of studies with missing data to estimate effect sizes interpreted using Cohen’s guidelines of small (*d >* 0.2), medium (*d >* 0.5), and large (*d >* 0.8) effect [77] would have been contacted to inform this. Post-hoc power was not reported in the studies, and a priori power calculations were minimal due to the nature of pilot and small studies, increasing risk of type I and II errors [53,92]. This may account for the varying findings and cautious interpretations across studies.

### 3.9. Within-Group Findings

Within-group and between-group findings differed in two studies [52,84]. Impellizzeri et al. (2024) [52] found some subtests showed significant improvement for cognitive function following MT, whereas within-group MT results were consistently significant. Similarly, Chou et al. (2024) [84] found between-group cognitive function non-significant, whereas within-group findings were significant following MT. The increased significance for within-group MT findings indicates an effect of MT, but may not be more effective than the comparator, suggesting reduced power and increased risk of type II error [93]. Therefore, within-group significant effects cannot be used to solely conclude the success of MT [78,93].

### 3.10. Between-Group Findings

Between-group data comparing groups were used to indicate direction of effect (Table S3) [78,79]. The Haire et al. study (2021) [55] was not included in the synthesis for direction of effect due to all groups including MT, hence there were no relevant between-group data. The Impellizzeri et al. (2020) [82] and Lee et al. (2024) [54] studies were also excluded from the data synthesis due to between-group comparisons being based on within-group data, rather than comparative statistical analysis.

**Cognitive Function.** Cognitive function was measured across all included neurological conditions, increasing heterogeneity for measures, population, and intervention, which may explain inconsistent findings (Table 3). Some aspects of cognitive function significantly improved following MT for PD and TBI, whereas no effect was found for stroke despite very small or no effect size (*d* = 0.00) [52,53,84]. One study used virtual reality (VR) MT interventions, potentially confounding comparability to non-VR MT, but remained included in the data synthesis because the comparison group also received VR, therefore controlled for [52].

In contrast, cognitive function significantly improved for the comparator group compared to the MT group in HD, suggesting MT is safe but recreational therapy (comparator) was more effective for improving cognitive function [56,58]. Alternatively, this may be attributed to the MT group having lower baseline cognitive function than the comparator, although not deemed a confounding factor, or the varying nature of advanced HD participants included [56].

**Mood.** Mood was measured using a variety of measures including the Hospital Anxiety and Depression Scale (HADS), Beck Depression Inventory-II, and Profile of Mood State (POMS) [11,33,84]. Following MT intervention there was no effect found for stroke, but small effect sizes (*d* = 0.02, 0.04 for depression and *d* = 0.24 for anxiety) [11,33,84]. This reflects participants’ variability, small sample sizes, and varying measures that have enabled an effect not to be found. Additionally, the measures have been validated in Taiwan and Italy, but should be interpreted with caution when measuring anxiety and depression separately with the HADS, and translating the POMS into Spanish, where meaning of constructs may differ, suggesting conclusions cannot be drawn reliably [11,33,49,88,94,95].

**Emotion.** Emotion was measured for one study researching stroke participants, finding significant improvements in emotional well-being following MT intervention with a medium effect size (*d* = 0.69) post-intervention [33]. A non-significant result was found at three-months follow-up, with very small effect size, however (*d* = 0.10), suggesting the improvement may not have sustained but the difference is negligible [33]. These were estimated based on a subtest result, rather than overall test battery conclusions from the Stroke Impact Scale [33]. Single subtests can be interpreted effectively to measure change, but further analysis is required to establish effect and impact [96].

**Behaviour.** Change in behaviour was measured by one study, observing no significant effect of MT for HD [56]. The results may differ if compared to treatment-as-usual rather than an alternative therapy [97,98]. However, the behaviour measures may not be sensitive to changes for the advanced HD participants [56]. Therefore, concluding the effect of MT cannot be confirmed.

**Quality of Life.** Quality of life found significant improvement for MS and post-stroke [11,57]. However, one study based its findings on subtests rather than overall measures [57]. Therefore, effect size is not known, and the significant findings reported do not represent the breadth of the outcome [57,99]. The estimated effect size for the Raglio et al. (2017) [11] finding was calculated as small (*d* = 0.18), indicating reduced applicability in practice.

## 4. Certainty of Evidence Using GRADE

### 4.1. Risk of Bias

The risk of bias increases for outcomes using self-report measures, such as mood and quality of life [75]. Behaviour is more likely assessed using observer-reported judgement, increasing bias except when assessors are blinded to allocation, whereas cognitive function is more likely assessed objectively, minimising bias [75,100].

There is potential bias from type of MT and its impact on an outcome. For example, Chou et al. (2024) [84] used speech and language-related NMT, whereas Raglio et al. (2017) [11] used relational NMT that facilitates rapport between the therapist and participant [101]. Both studies measured mood outcomes; however, the interventions approached different intentions, suggesting this heterogeneity may cause the outcomes from different approaches improving mood with differing effects.

### 4.2. Inconsistency

Statistical measures such as I^2^ or Cochran’s *p*-value cannot be used to establish consistency due to heterogenic data [102]. However, inconsistencies in effect direction can be explained by the known variation across PICOS and the likely lack of power due to small sample sizes across studies reducing the likelihood of finding effect [48,50,81,93].

### 4.3. Indirectness

The MT interventions used across studies are evidence-based NMT techniques and protocols applicable to clinical practice, except Impellizzeri et al. (2024) [52], which incorporated VR into MT, less widely implemented clinically. Similarly, the quantity of intervention varied, such as Chou et al. (2024) [84] receiving MT extra to treatment-as-usual, which may confound findings by suggesting input intensity influences effect rather than MT. Alternatively, Impellizzeri et al. (2020) [82] replaced half of the treatment-as-usual therapy with MT, as recommended by NICE (2023) [43]. This increases applicability to settings where funding for MT replaces treatment-as-usual, rather than added [7].

The neurological conditions studied reflect service provision that may consider including MT into the multi-disciplinary team [31]. The treatment-as-usual comparators reflect clinical practice, and no study compared MT to no treatment, deemed unrealistic and unethical [97,98,103].

### 4.4. Imprecision

Confidence intervals were not available to clearly determine the certainty of precision across studies. However, in Chou et al. (2024) [84], the confidence interval for cognitive function and mood between-groups is much wider than the mean difference, and span positive and negative values, indicating reduced certainty of effect. Sample heterogeneity and size reduces statistical power and therefore precision.

Precision cannot be determined from power due to lack of reporting. Four of the ten studies are pilots, less likely to meet statistical power than main studies; however main study sample sizes remained small, with a pilot study obtaining the largest sample included [84,92]. Power calculations were carried out a priori for three studies, where Chou et al. (2024) [84] had a sample size that met the criterion, but Siponkoski et al. (2020) and Van Bruggen-Rufi et al. (2017) [53,56] criteria were not sustained due to drop-out rates, reducing precision.

### 4.5. Publication Bias

Magnitude of effect was not clear, risking inflated conclusions, particularly given effect sizes were small where available [11,55,81,84]. Furthermore, main studies did not differ in precision or sample size from pilots, risking exaggerated generalisability from reduced certainty.

### 4.6. Overall Certainty of Evidence and Importance of Outcome

The overall certainty of evidence was low, which can be attributed to the limited reporting of effect size, small sample sizes, and heterogeneity within the elements of PICOS [48,50]. This outcome suggests findings should be interpreted with caution but does not disregard the potential effects of MT on psychological outcomes for neurological conditions, given the limitations in the consistency, precision, and bias within and between the studies [58].

## 5. Discussion

This systematic review aimed to establish whether there is an effect of MT on psychological outcomes for neurological conditions. In summary, cognitive function was measured across all included neurological conditions in this review, showing MT improved cognitive function in PD and TBI, but not for stroke or HD, with HD showing improvement in the comparator group [33,52,53,56,84]. A significant improvement was found for emotional well-being, but not mood following MT for people who had stroke [33,84]. Behaviour was not found to significantly improve following MT in HD. However, quality of life improved in MS and stroke following MT [11,56,57].

All studies included randomised participants. However, participants and therapists could not be blinded from allocation, thus increasing risk of bias. Missing outcome data were accounted for across studies, and standardised measures were used across studies, but not always valid and reliable for the population studied, again increasing risk of bias. Studies often limited reporting to significant findings, and had limited transparency between planned and actual analyses, suggesting optimism bias [90]. One study indicated high risk [84], and one low risk [53], with the remaining eight studies having some concerns of bias (Table S5).

The GRADE discussion suggests reduced certainty due to risk of bias increasing for self-reported outcomes but is improved through assessor blinding. Consistency of direction of effect is limited by heterogeneity across all PICOS elements, despite being relevant to clinical practice, improving directness [48,50]. Precision is reduced with wide confidence intervals when provided and no potential power differences between pilot and main studies. Publication bias may be impacted by inflated conclusions from lack of effect size reporting.

The findings in this review are similar to previous reviews. MT is perceived as a positive intervention that has potential impact on psychological outcomes, but PICOS heterogeneity reduces findings of group difference and certainty of findings, which are limitations across MT research [6,39,48,50,65]. Neurological conditions range across studies, with differing severities, from different countries. This heterogeneity means that consideration for cultural applicability of the MT intervention and the meaning and translation validity of outcome measures, including specific subtests, is required [17,104]. Similarly, the definition of “treatment-as-usual” for comparator groups is rarely clarified, suggesting there may be variations in the comparator group and the extent of similarity to the intervention, potentially reducing certainty of results [105,106].

The current review is limited in synthesising outcomes using vote counting for direction of effect [24]. It does not account for extent or magnitude of effect, so the risk of type I or II error is uncertain, and also limits the applicability of GRADE to assess certainty of outcomes [24]. Similarly, not all studies were included in the data synthesis due to lack of reporting of between-group results and homogeneity between treatment intervention and comparator intervention [54,55,82]. This reduces an already small number of studies included, reducing the value in vote counting direction of effect, since proportion of effects could not be determined reliably. Therefore, there is reduced generalisability of findings and certainty across studies.

The limited papers found for this review are an attempt to reduce heterogeneity by narrowing MT practice, rather than general music-based interventions, but still presents a range of interventions [65]. However, narrowing the intervention criteria to specific NMT may not account for the diversity of methods and symptoms within disorders to which MT can apply [37,48,50].

Despite recommendations for RCTs to more accurately determine the effectiveness of MT, the RCTs reviewed have limitations in quality and reporting [6,37,39,43]. Since the heterogenic nature of these studies cannot be controlled for without reducing clinical applicability, future research should focus on larger-scale RCTs to account for diversity, with comparison of MT to treatment-as-usual that is explicitly defined and distinguished from the intervention [29,39,107]. MT is well established for dementia compared to other neurological conditions, enabling increased access to participants [29]. Hence, funding is required to enable MT to be established for large-scale RCT research to be conducted.

Considering the limited quality of evidence and reporting of findings, it has been established that MT has potential for positive effects on psychological outcomes. However, the certainty of findings is low and, therefore, conclusions about MT effect on psychological outcomes for neurological conditions cannot be drawn.

## 6. Conclusions

This review demonstrates the potential effect of MT to improve psychological outcomes including cognitive function, mood, emotion, behaviour, and quality of life, for people living with neurological conditions. RCTs are limited in certainty and bias due to the heterogenic nature of MT, neurological conditions, and measures of psychological outcomes, but often carried out on a small scale.

MT has the potential to be a cost-effective, safe, and valuable intervention that could minimise unnecessary pharmacotherapy [15,58]. Future research should endeavour to define and measure the interventions and corresponding effects on a large scale, to better inform intervention outcomes and prevent missed opportunity that MT could offer.

## Figures and Tables

**Table 1:** Outcome variables and how they are defined by the included studies.

Outcome Variable	Definition for Included Studies
Cognitive function	Executive functions, memory, visuospatial abilities, attention, communication [52,53]
Mood	Depression, anxiety, anger, vigour, fatigue [11,33,54]
Emotion	Self-perceived emotional well-being, emotional awareness of self and others, sharing of emotions [33,52]
Self-efficacy	Sense of competence in managing new and challenging situations [55]
Behaviour	Communication and expressive skills, mental rigidity, aggression [56]
Affect	Valence, arousal, dominance [55]
Quality of life	An individual’s perception of their physical and mental state, and social position [57]

**Table 2:** Criteria used to determine eligible studies for inclusion in the review.

Inclusion Criteria	Exclusion Criteria
(a) Peer-reviewed original empirical RCTs	(a) Studies with dementia or spinal cord injury
(b) Adults with neurological conditions	(b) Studies with neurodevelopmental conditions
(c) MT delivered by a board-certified music therapist or a therapist skilled in delivering specific evidence-based NMT	(c) Study designs such as review, protocol, or feasibility studies
(d) Published between 1 January 2015–31 January 2025,	(d) Studies with qualitative or mixed methods data
(e) Published in the English language	(e) Studies containing non-psychological outcomes

## Data Availability

The authors confirm that the data generated from this study are available both within the article as well as the accompanying Supplementary Material.

## Supplementary Materials

The following supporting information can be downloaded at: https://www.mdpi.com/article/10.3390/medicina61091611/s1, Table S1. Search terms inputted into the four databases; Table S2. Overview of synthesis and summary of findings from included studies; Table S3. Effect direction plot for between-group effects of psychological outcomes for neurological condition; Table S4. PRISMA guidelines; Table S5. Cochrane Risk of Bias (RoB) 2 summary.

## References

[1] Devlin K., Alshaikh J.T., Pantelyat A. (2019). Music therapy and music-based interventions for movement disorders. Curr. Neurol. Neurosci. Rep..

[2] De Witte M., Pinho A.D.S., Stams G.J., Moonen X., Bos A.E., Van Hooren S. (2022). Music therapy for stress reduction: A systematic review and meta-analysis. Health Psychol. Rev..

[3] Hurkmans J., de Bruijn M., Boonstra A.M., Jonkers R., Bastiaanse R., Arendzen H., Reinders-Messelink H.A. (2012). Music in the treatment of neurological language and speech disorders: A systematic review. Aphasiology.

[4] Koelsch S. (2009). A neuroscientific perspective on music therapy. Ann. N. Y. Acad. Sci..

[5] Galińska E. (2015). Music therapy in neurological rehabilitation settings. Psychiatr Pol..

[6] Robb S.L., Hanson-Abromeit D., May L., Hernandez-Ruiz E., Allison M., Beloat A., Daughtery S., Kurtz R., Ott A., Oyedele O.O. (2018). Reporting quality of music intervention research in healthcare: A systematic review. Complement. Ther. Med..

[7] Grau-Sánchez J., Jamey K., Paraskevopoulos E., Dalla Bella S., Gold C., Schlaug G., Belleville S., Rodríguez-Fornells A., Hackney M.E., Särkämö T. (2022). Putting music to trial: Consensus on key methodological challenges investigating music-based rehabilitation. Ann. N. Y. Acad. Sci..

[8] Howlett J.R., Nelson L.D., Stein M.B. (2022). Mental health consequences of traumatic brain injury. Biol. Psychiatry.

[9] McCaffrey T. (2018). Evaluating music therapy in adult mental health services: Tuning into service user perspectives. Nord. J. Music. Ther..

[10] Moore K.S. (2013). A systematic review on the neural effects of music on emotion regulation: Implications for music therapy practice. J. Music. Ther..

[11] Raglio A., Zaliani A., Baiardi P., Bossi D., Sguazzin C., Capodaglio E., Imbriani C., Gontero G., Imbriani M. (2017). Active music therapy approach for stroke patients in the post-acute rehabilitation. Neurol. Sci..

[12] Thompson N., Iyemere K., Underwood B.R., Odell-Miller H. (2023). Investigating the impact of music therapy on two in-patient psychiatric wards for people living with dementia: Retrospective observational study. BJPsych Open.

[13] Wilson L., Horton L., Kunzmann K., Sahakian B.J., Newcombe V.F., Stamatakis E.A., von Steinbuechel N., Cunitz K., Covic A., Maas A. (2021). Understanding the relationship between cognitive performance and function in daily life after traumatic brain injury. J. Neurol. Neurosurg. Psychiatry.

[14] Lena F., Modugno N., Greco G., Torre M., Cesarano S., Santilli M., Abdullahi A., Giovannico G., Etoom M. (2023). Rehabilitation interventions for improving balance in Parkinson’s disease: A narrative review. Am. J. Phys. Med. Rehabil..

[15] Brancatisano O., Baird A., Thompson W.F. (2020). Why is music therapeutic for neurological disorders? The Therapeutic Music Capacities Model. Neurosci. Bobehav. Riv..

[16] Leins A.K., Spintge R., Hallam S., Cross I., Thaut M. (2008). Music therapy in medical and neurological rehabilitation settings. The Oxford Handbook of Music Psychology.

[17] Machado Sotomayor M.J., Arufe-Giráldez V., Ruíz-Rico G., Navarro-Patón R. (2021). Music therapy and Parkinson’s disease: A systematic review from 2015–2020. Int. J. Environ. Res. Public Health.

[18] Magee W.L. (2019). Why include music therapy in a neuro-rehabilitation team?. ACNR.

[19] Mercier L.J., Langelier D.M., Buchanan J., Robinson S., Plamondon S. (2024). Development and integration of a music therapy program in the neurologic inpatient setting: A qualitative study. Disabil. Rehabil..

[20] Murtaugh B., Morrissey A.M., Fager S., Knight H.E., Rushing J., Weaver J. (2024). Music, occupational, physical, and speech therapy interventions for patients in disorders of consciousness: An umbrella review. NeuroRehabilitation.

[21] Thaut M.H., Thaut M., Hoemberg V. (2014). Assessment and the transformational design model (TDM). Handbook of Neurologic Music Therapy.

[22] Breuer E., Lee L., De Silva M., Lund C. (2015). Using theory of change to design and evaluate public health interventions: A systematic review. Implement. Sci..

[23] de L’Etoile S.K., Hallam S., Cross I., Thaut M. (2014). Processes of music therapy: Clinical and scientific rationales and models. The Oxford handbook of music psychology.

[24] Campbell M., McKenzie J.E., Sowden A., Katikireddi S.V., Brennan S.E., Ellis S., Hartmann-Boyce J., Ryan R., Shepperd S., Thomas J. (2020). Synthesis without meta-analysis (SWiM) in systematic reviews: Reporting guideline. BMJ.

[25] Popay J., Roberts H., Sowden A., Petticrew M., Arai L., Rodgers M., Britten N., Roen K., Duffy S. (2006). Guidance on the Conduct of Narrative Synthesis in Systematic Reviews: A Product from the ESRC Methods Programme Version.

[26] Altenmüller E., Schlaug G. (2013). Neurologic music therapy: The beneficial effects of music making on neurorehabilitation. Acoust. Sci. Technol..

[27] Lam H.L., Li W.T.V., Laher I., Wong R.Y. (2020). Effects of music therapy on patients with dementia—A systematic review. Geriatrics.

[28] Lanb L.C.L.S.H., Lanc S.J., Hsiehe Y.P. (2024). Effectiveness of the Music Therapy in Dementia: A Systematic Review and Meta-Analysis of Randomized Controlled Trials. Dement. Geriatr. Cogn. Disord..

[29] Moreno-Morales C., Calero R., Moreno-Morales P., Pintado C. (2020). Music therapy in the treatment of dementia: A systematic review and meta-analysis. Front. Med..

[30] National Institute for Health and Care Excellence (NICE) (2019). Dementia Quality Standard [NICE Guideline Quality Standard No. 184]. https://www.nice.org.uk/guidance/qs184.

[31] NHS England (2014). NHS Standard Contract for Specialised Rehabilitation for Patients with Highly Complex Needs (All Ages). https://www.england.nhs.uk/wp-content/uploads/2014/04/d02-rehab-pat-high-needs-0414.pdf.

[32] Thompson N., Odell-Miller H. (2024). An audit of music therapy in acute National Health Service (NHS) settings for people with dementia in the UK and adaptations made due to COVID-19. Approaches Interdiscip. J. Music. Ther..

[33] Segura E., Grau-Sánchez J., Cerda-Company X., Porto M.F., De la Cruz-Puebla M., Sanchez-Pinsach D., Cerquides J., Duarte E., Palumbo A., Turry A. (2024). Enriched music-supported therapy for individuals with chronic stroke: A randomized controlled trial. J. Neurol..

[34] Fusar-Poli L., Bieleninik Ł., Brondino N., Chen X.J., Gold C. (2018). The effect of music therapy on cognitive functions in patients with dementia: A systematic review and meta-analysis. Aging Ment. Health.

[35] Zaatar M.T., Alhakim K., Enayeh M., Tamer R. (2023). The transformative power of music: Insights into neuroplasticity, health, and disease. Brain Behav. Immun.-Health.

[36] Chen W.G., Iversen J.R., Kao M.H., Loui P., Patel A.D., Zatorre R.J., Edwards E. (2022). Music and Brain Circuitry: Strategies for Strengthening Evidence-Based Research for Music-Based Interventions. J. Neurosci..

[37] Freitas C., Fernández-Company J.F., Pita M.F., Garcia-Rodriguez M. (2022). Music therapy for adolescents with psychiatric disorders: An overview. Clin. Child. Psychol. Psychiatry.

[38] Tramontano M., De Angelis S., Mastrogiacomo S., Princi A.A., Ciancarelli I., Frizziero A., Iosa M., Paolucci S., Morone G. (2021). Music-based techniques and related devices in neurorehabilitation: A scoping review. Expert. Rev. Med. Devices.

[39] Mishra R., Florez-Perdomo W.A., Shrivatava A., Chouksey P., Raj S., Moscote-Salazar L.R., Rahman M.M., Sutar R., Agrawal A. (2021). Role of music therapy in traumatic brain injury: A systematic review and meta-analysis. World Neurosurg..

[40] Odell-Miller H. (2016). The role, function and identity of music therapists in the 21st century, including new research and thinking from a UK perspective. BJMT.

[41] Carr C.E., Tsiris G., Swijghuisen Reigersberg M. (2017). Understanding the present, re-visioning the future: An initial mapping of music therapists in the United Kingdom. BJMT.

[42] Wood J., Sandford S., Bailey E. (2016). ‘The whole is greater’. Developing music therapy services in the National Health Service: A case study revisited. BJMT.

[43] National Institute for Health and Care Excellence (NICE) (2023). Stroke Rehabilitation in Adults [NICE Guideline No. 236]. https://www.nice.org.uk/guidance/ng236/chapter/Recommendations.

[44] Hariton E., Locascio J.J. (2018). Randomised controlled trials—The gold standard for effectiveness research. BJOG.

[45] García-Navarro E.B., Buzón-Pérez A., Cabillas-Romero M. (2022). Effect of Music Therapy as a Non-Pharmacological Measure Applied to Alzheimer’s Disease Patients: A Systematic Review. Nurs. Rep..

[46] Bleibel M., El Cheikh A., Sadier N.S., Abou-Abbas L. (2023). The effect of music therapy on cognitive functions in patients with Alzheimer’s disease: A systematic review of randomized controlled trials. Alz Res. Ther..

[47] Falzon L., Davidson K.W., Bruns D. (2010). Evidence searching for evidence-based psychology practice. Prof. Psychol. Res. Pract..

[48] Higgins J.P.T., Green S. (2011). Cochrane Handbook for Systematic Reviews of Interventions.

[49] Chiu E.C., Chen Y.J., Wu W.C., Chou C.X., Yu M.Y. (2022). Psychometric comparisons of three depression measures for patients with stroke. AJOT.

[50] Nishikawa-Pacher A. (2022). Research questions with PICO: A universal mnemonic. Publications.

[51] Richardson W.S., Wilson M.C., Nishikawa J., Hayward R.S. (1995). The well-built clinical question: A key to evidence-based decisions. ACP J. Club.

[52] Impellizzeri F., Maggio M.G., De Pasquale P., Bonanno M., Bonanno L., De Luca R., Paladina G., Alibrandi A., Milardi D., Thaut M. (2024). Coupling neurologic music therapy with immersive virtual reality to improve executive functions in individuals with Parkinson’s disease: A Quasi-Randomized Clinical Trial. Clin. Park. Relat. Disord..

[53] Siponkoski S.T., Martínez-Molina N., Kuusela L., Laitinen S., Holma M., Ahlfors M., Jordan-Kilkki P., Ala-Kauhaluoma K., Melkas S., Pekkola J. (2020). Music therapy enhances executive functions and prefrontal structural neuroplasticity after traumatic brain injury: Evidence from a randomized controlled trial. J. Neurotrauma.

[54] Lee S.J., Dvorak A.L., Manternach J.N. (2024). Therapeutic Singing and Semi-Occluded Vocal Tract Exercises for Individuals with Parkinson’s Disease: A Randomized Controlled Trial of a Single Session Intervention. J. Music. Ther..

[55] Haire C.M., Vuong V., Tremblay L., Patterson K.K., Chen J.L., Thaut M.H. (2021). Effects of therapeutic instrumental music performance and motor imagery on chronic post-stroke cognition and affect: A randomized controlled trial. NeuroRehabilitation.

[56] van Bruggen-Rufi M.C., Vink A.C., Wolterbeek R., Achterberg W.P., Roos R.A. (2017). The effect of music therapy in patients with Huntington’s disease: A randomized controlled trial. J. Huntington’s Dis..

[57] Poćwierz-Marciniak I., Bidzan M. (2017). The influence of music therapy on quality of life after a stroke. Health Psychol. Rep..

[58] Blackburn R., Bradshaw T. (2014). Music therapy for service users with dementia: A critical review of the literature. J. Psychiatr. Men. Health Nurs..

[59] Hoffecker L. (2020). Grey Literature Searching for Systematic Reviews in the Health Sciences. Ser. Libr..

[60] Kelly J., Sadeghieh T., Adeli K. (2014). Peer review in scientific publications: Benefits, critiques, & a survival guide. J. Int. Fed. Clin. Chem. Lab. Med..

[61] Barrington A. (2015). Perspectives on the development of the music therapy profession in the UK. Approaches.

[62] British Association for Music Therapy (2020). Guidelines on Professional Titles for Music Therapists.

[63] Chandler G., Maclean E. (2024). “There has probably never been a more important time to be a music therapist”: Exploring how three music therapy practitioners working in adult mental health settings in the UK experienced the first year of the COVID-19 pandemic. Approaches.

[64] Helbach J., Pieper D., Mathes T., Rombey T., Zeeb H., Allers K., Hoffmann F. (2022). Restrictions and their reporting in systematic reviews of effectiveness: An observational study. BMC Med. Res. Methodol..

[65] Kamioka H., Tsutani K., Yamada M., Park H., Okuizumi H., Tsuruoka K., Honda T., Okada S., Park S., Kitayuguchi J. (2014). Effectiveness of music therapy: A summary of systematic reviews based on randomized controlled trials of music interventions. Patient Prefer. Adherence.

[66] Pieper D., Puljak L. (2021). Language restrictions in systematic reviews should not be imposed in the search strategy but in the eligibility criteria if necessary. J. Clin. Epidemiol..

[67] Bond C., Lancaster G.A., Campbell M., Chan C., Eddy S., Hopewell S., Mellor K., Thabane L., Eldridge S. (2023). Pilot and feasibility studies: Extending the conceptual framework. PFS.

[68] Arain M., Campbell M.J., Cooper C.L., Lancaster G.A. (2010). What is a pilot or feasibility study? A review of current practice and editorial policy. BMC Med. Res. Methodol..

[69] Mayer-Benarous H., Benarous X., Vonthron F., Cohen D. (2021). Music therapy for children with autistic spectrum disorder and/or other neurodevelopmental disorders: A systematic review. Front. Psychiatry.

[70] Ahuja C.S., Wilson J.R., Nori S., Kotter M., Druschel C., Curt A., Fehlings M.G. (2017). Traumatic spinal cord injury. Nat. Rev. Dis. Primers.

[71] Guyatt G.H., Oxman A.D., Vist G.E., Kunz R., Falck-Ytter Y., Alonso-Coello P., Schünemann H.J. (2008). GRADE: An emerging consensus on rating quality of evidence and strength of recommendations. BMJ.

[72] Page M.J., McKenzie J.E., Bossuyt P.M., Boutron I., Hoffmann T.C., Mulrow C.D., Shamseer L., Tetzlaff J.M., Akl E.A., Brennan S.E. (2021). The PRISMA 2020 statement: An updated guideline for reporting systematic reviews. BMJ.

[73] Higgins J.P., Savović J., Page M.J., Elbers R.G., Sterne J.A., Higgins J.P.T., Thomas J., Chandler J., Cumpston M., Li T., Page M.J., Welch V.A. (2019). Assessing risk of bias in a randomized trial. Cochrane Handbook for Systematic Reviews of Interventions.

[74] Nejadghaderi S.A., Balibegloo M., Rezaei N. (2024). The Cochrane risk of bias assessment tool 2 (RoB 2) versus the original RoB: A perspective on the pros and cons. Health Sci. Rep..

[75] Sterne J.A.C., Savović J., Page M.J., Elbers R.G., Blencowe N.S., Boutron I., Cates C.J., Cheng H.Y., Corbett M.S., Eldridge S.M. (2019). RoB 2: A revised tool for assessing risk of bias in randomised trials. BMJ.

[76] Thomson H.J., Thomas S. (2013). The effect direction plot: Visual display of non-standardised effects across multiple outcome domains. Res. Synth. Methods.

[77] Cohen J. (1992). Quantitative methods in psychology: A power primer. Psychol. Bull..

[78] Bauchner H., Golub R.M., Fontanarosa P.B. (2019). Reporting and interpretation of randomized clinical trials. JAMA.

[79] Bhide A., Shah P.S., Acharya G. (2018). A simplified guide to randomized controlled trials. AOGS.

[80] Murad M.H., Mustafa R.A., Schünemann H.J., Sultan S., Santesso N. (2017). Rating the certainty in evidence in the absence of a single estimate of effect. BMJ EBM.

[81] Schünemann H.J., Higgins J.P., Vist G.E., Glasziou P., Akl E.A., Skoetz N., Guyatt G.H., Higgins J.P.T., Thomas J., Chandler J., Cumpston M., Li T., Page M.J., Welch V.A. (2019). Completing ‘summary of findings’ tables and grading the certainty of the evidence. Cochrane Handbook for Systematic Reviews of Interventions.

[82] Impellizzeri F., Leonardi S., Latella D., Maggio M.G., Foti Cuzzola M., Russo M., Sessa E., Bramanti P., De Luca R., Calabrò R.S. (2020). An integrative cognitive rehabilitation using neurologic music therapy in multiple sclerosis: A pilot study. Medicine (Baltimore)..

[83] Estellat C., Torgerson D.J., Ravaud P. (2009). How to perform a critical analysis of a randomised controlled trial. Best. Pract. Res. Clin. Rheumatol..

[84] Chou C.H., Chen P.C., Huang Y.C., Yang T.H., Wang L.Y., Chen I.H., Lee H.J., Lee Y.Y. (2024). Neurological music therapy for poststroke depression, activity of daily living and cognitive function: A pilot randomized controlled study. Nord. J. Music. Ther..

[85] Van Ginkel J.R., Linting M., Rippe R.C., Van Der Voort A. (2020). Rebutting existing misconceptions about multiple imputation as a method for handling missing data. J. Pers. Assess..

[86] Van Buuren S., Groothuis-Oudshoorn K. (2011). MICE: Multivariate imputation by chained equations in R. J. Stat. Softw..

[87] Austin P.C., White I.R., Lee D.S., van Buuren S. (2021). Missing data in clinical research: A tutorial on multiple imputation. Can. J. Cardiol..

[88] Andrade E., Arce C., Torrado J., Garrido J., De Francisco C., Arce I. (2010). Factor structure and invariance of the POMS mood state questionnaire in Spanish. Span. J. Psychol..

[89] Boutron I., Dutton S., Ravaud P., Altman D.G. (2010). Reporting and interpretation of randomized controlled trials with statistically nonsignificant results for primary outcomes. JAMA.

[90] Carter E.C., Schönbrodt F.D., Gervais W.M., Hilgard J. (2019). Correcting for bias in psychology: A comparison of meta-analytic methods. Adv. Meth Pract. Psychol. Sci..

[91] van Bruggen-Rufi M., Vink A., Achterberg W., Roos R. (2016). Music therapy in Huntington’s disease: A protocol for a multi-center randomized controlled trial. BMC Psychol..

[92] Kraemer H.C., Mintz J., Noda A., Tinklenberg J., Yesavage J.A. (2006). Caution regarding the use of pilot studies to guide power calculations for study proposals. Arch. Gen. Psychiatry.

[93] Beato M. (2022). Recommendations for the design of randomized controlled trials in strength and conditioning. Common. Des. Data interpretation. Front. Sports Act. Living.

[94] Annunziata M.A., Muzzatti B., Bidoli E., Flaiban C., Bomben F., Piccinin M., Gipponi K.M., Mariutti G., Busato S., Mella S. (2020). Hospital Anxiety and Depression Scale (HADS) accuracy in cancer patients. Support. Care Cancer.

[95] Costantini M., Musso M., Viterbori P., Bonci F., Del Mastro L., Garrone O., MVenturini M., Morasso G. (1999). Detecting psychological distress in cancer patients: Validity of the Italian version of the Hospital Anxiety and Depression Scale. Support Care Cancer.

[96] Annell S., Sjöberg A., Sverke M. (2014). Use and interpretation of test scores from limited cognitive test batteries: How g+ Gc can equal g. Scand. J. Psychol..

[97] Essers B., Veerbeek J.M., Luft A.R., Verheyden G. (2024). The feasibility of the adapted H-GRASP program for perceived and actual daily-life upper limb activity in the chronic phase post-stroke. Disabil. Rehabil..

[98] Simpson L.A., Eng J.J., Chan M. (2017). H-GRASP: The feasibility of an upper limb home exercise program monitored by phone for individuals post stroke. Disabil. Rehabil..

[99] Russell E.W., Russell S.L., Hill B.D. (2005). The fundamental psychometric status of neuropsychological batteries. Arch. Clin. Neuropsychol..

[100] Casaletto K.B., Heaton R.K. (2017). Neuropsychological assessment: Past and future. JNS.

[101] Thaut M., Hoemberg V. (2014). Handbook of Neurologic Music Therapy.

[102] von Hippel P.T. (2015). The heterogeneity statistic I 2 can be biased in small meta-analyses. BMC Med Res. Methodol..

[103] Königs M., Beurskens E.A., Snoep L., Scherder E.J., Oosterlaan J. (2018). Effects of timing and intensity of neurorehabilitation on functional outcome after traumatic brain injury: A systematic review and meta-analysis. Arch. Phys. Med. Rehabil..

[104] Kern P., Tague D.B. (2017). Music therapy practice status and trends worldwide: An international survey study. J. Music. Ther..

[105] Dobran S.A., Gherman A. (2024). Neurorehabilitation across continents: The WFNR-EFNR regional meeting in conjunction with the 19th congress of the society for the study of neuroprotection and neuroplasticity and the 19th international summer school of neurology in Baku, Azerbaijan. J. Med. Life.

[106] Nasios G., Messinis L., Dardiotis E., Sgantzos M. (2023). Neurorehabilitation: Looking Back and Moving Forward. Healthcare.

[107] Fernainy P., Cohen A.A., Murray E., Losina E., Lamontagne F., Sourial N. (2024). Rethinking the pros and cons of randomized controlled trials and observational studies in the era of big data and advanced methods: A panel discussion. BMC Proc..

