# Supplementary material for: The Effect of Music Therapy on Psychological Outcomes for Neurological Conditions: A Systematic Review
Source: Medicina (Kaunas). 2025 Sep 5;61(9):1611. doi: 10.3390/medicina61091611 (PMC12471332; doi:10.3390/medicina61091611)
Supplement: Supplementary file 1 [file medicina-61-01611-s001.zip › medicina-3837967-supplementary.pdf]

Table S1. Search terms inputted into the four databases.

Search terms for PubMed and Scopus:<br><br>[“music* therap*”] AND [neurorehab* OR neuro-rehab* OR “neuro* rehab*” OR neurologic* OR “neurologic* condition*” OR “neurologic* disorder*” OR Parkinson* OR “brain injur*” OR TBI OR “traumatic brain injur*” OR ABI OR “acquired brain injur*” OR “brain tumour*” OR “brain tumor*” OR “multiple sclerosis” OR MS OR stroke* OR encephal* OR epileps* OR “motor neurone disease*” OR MND OR Huntington* OR HD OR “disorder* of conscious*” OR DOC OR “minimal* conscious*” OR vegetative OR “unresponsive wakeful* syndrome*”]
Search terms for PsycInfo and CINAHL:<br><br>[music* therap*] AND [neurorehab* OR neuro-rehab* OR neuro* rehab* OR neurologic* OR neurologic* condition* OR neurologic* disorder* OR Parkinson* OR brain injur* OR TBI OR traumatic brain injur* OR ABI OR acquired brain injur* OR brain tumour* OR brain tumor* OR multiple sclerosis OR MS OR stroke* OR encephal* OR epileps* OR motor neurone disease* OR MND OR Huntington* OR HD OR disorder* of conscious* OR DOC OR minimal* conscious* OR vegetative OR unresponsive wakeful* syndrome*]

Note. Search fields were limited to title and abstract for all databases, with the addition of keywords in PsycINFO and Scopus.

Search terms were identified from previous literature and neurorehabilitation service provisions, alongside support from a specialist librarian at the University of Leicester.

Table S2. Overview of synthesis and summary of findings from included studies.

	Neurological Condition	Psychological Outcome	Reported Effect Direction	Effect Size (Cohen's *d*)	*p*-Value	Mean Difference [95% CI]
Chou et al. (2024) [84]	Stroke	Cognitive function	↑<br>→	Not reported.<br>Calculated as 0.40 (within-group intervention), 0.00 (between-group)	<0.001 (within-group)<br>0.355 (between group)	1.04 [0.51–1.57]<br>0.02 [−2.88–2.83]
		Mood (depression)	→<br>→	Not reported.<br>Calculated as 0.16 (within-group intervention),<br>0.02 (between-groups)	0.129 (within-group)<br>0.740 (between-group)	−0.84 [−1.93–0.25]<br>−0.05 [−2.80–2.90]
Haire et al. (2021) [55]	Stroke	Cognitive function	→	Not reported.<br>Trail-making test:<br>Calculated as 0.12 (within-group MT only)<br>0.27 (within-group MT + cMI)<br>0.27 (within-group MT + MI)<br>Digit span test:<br>Calculated as 0.09 (within-group MT only)<br>0.00 (within-group MT + cMI)<br>0.10 (within-group MT + MI)	Trail-making test:<br>1.00 (MT only)<br>0.125 (MT + cMI)<br><0.05 (MT + MI)<br>Digit span test:<br>0.459 (MT only)<br>0.865 (MT + cMI)<br>0.669 (MT + MI)	Not reported
		Affect	→	Not reported.<br>Calculated as 0.34 (within-group MT only)<br>0.40 (within-group MT + cMI)<br>0.47 (within-group MT + MI)	0.105 (MT only)<br><0.05 (MT + cMI)<br>0.261 (MT + MI)	Not reported
		Mood	→	Not reported. Calculated as 0.28 (within-group MT only)<br>0.90 (within-group MT + cMI)<br>0.05 (within-group MT + MI)	0.147 (MT only)<br><0.05 (MT + cMI)<br>0.492 (MT + MI)	Not reported

		Self-efficacy	→	Not reported. Calculated as 0.21 (within-group MT only)<br>0.35 (within-group MT + cMI)<br>0.02 (within-group MT + MI)	0.202 (MT only)<br>0.098 (MT + cMI)<br>1.00 (MT + MI)	Not reported
Poćwierz-Marciniak & Bidzan (2017) [57]	Stroke	Quality of life	↑ *	Not reported. Not calculated due to multiple measures used for outcome.	<0.05 (between-groups) for some scales.	Not reported
Raglio et al. (2017) [11]	Stroke	Quality of life	↑	Not reported. Calculated as 0.18 (between-group)	0.19 (between-group)	Not reported
		Mood (depression)	↑	Not reported. Calculated as 0.04 (between-group)	<0.05 (between-group)	Not reported
		Mood (anxiety)	→	Not reported.<br>Calculated as 0.24 (between-group)	0.25 (between-group)	Not reported
Segura et al. (2024) [33]		Cognitive functions	→ *	Not reported. Not calculated due to volume of measures for outcome.	>0.05 on subscales (between-group) at post-intervention or follow-up.	Refer to original paper, not reported here due to volume of measures used.
	Stroke	Emotion	↑ *	Not reported. Not calculated as 0.69 (between-group) post-intervention, 0.10 (between-group) at follow-up.	<0.05 subscale (between-group) at post-intervention. Not significant at follow-up.	Refer to original paper, not reported here due to volume of measures used.
		Mood	↑ *	Not reported. Not calculated due to volume of measures for outcome.	<0.05 on one subscale (between-group) at post-intervention. Not significant at follow-up.	Refer to original paper, not reported here due to volume of measures used.

Impellizzeri et al. (2020) [82]	Multiple sclerosis	Cognitive function	↑ *<br>↑ *	Not reported. Not enough information provided (no standard deviation).	Overall battery outcome not reported. 4/9 subscales <0.05 within-group experimental) otherwise non-significant.	No overall outcome results provided.
		Mood (depression)	↑<br>↑	Not reported. Not enough information provided (no standard deviation).	<0.05 (within-group experimental)<br>0.278 (within-group control).	5.60 [3.7–7.72]<br>−0.66 [−1.93–0.60]
		Emotion	↑ *<br>↑ *	Not reported. Not enough information provided (no standard deviation).<br>No overall outcome results provided.	<0.05 for all subscales (within-group experimental).	No overall outcome results provided.
		Quality of life	↑<br>↑	Not reported. Not enough information provided (no standard deviation).	<0.05 for all subscales (within-group experimental).	No overall outcome results provided.
Impellizzeri et al. (2024) [52]	Parkinson's disease	Cognitive function	↑<br>↑ *	Not reported. Reported the median, skewed data effect sizes to be discussed with statistician if time allowed.	<0.05 (within-group experimental)<br><0.05 within some sub-scales (between- group).	Not reported for change scores.
Lee et al. (2024) [54]	Parkinson's disease	Mood	↑ *	Not reported. Not calculated due to multiple subtests for outcome.	<0.05 sad, anxious, angry (within-group)<br>0.127 happy (within-group)	Not reported
Siponkoski et al. (2020) [53]	Traumatic brain injury	Cognitive function	↑ *	Not reported. Executive function − η2p = 0.093 (medium-large).<br>Set-shifting − η2p = 0.112 (medium-large).<br>Note these are not directly comparable to Cohen's *d*.	<0.05 (between-group) for executive functions, >0.05 (between-group) set-shifting, >0.05 (between-group) for reasoning and verbal memory.	
Van Bruggen-Rufi et al. (2017) [56]	Huntington's disease	Social-cognitive function	↓	Not reported. Not calculated due to limited data available.	<0.05 (between-groups)	2.88 [0.108–5.65]
		Behaviour	→	Not reported. Not calculated due to limited data available.	0.125 (between groups) on BOSH<br>0.630 (between groups on PBA-S	4.60 [−1.32–10.52]<br>−1.39 [−7.16–4.38]

↑ = music therapy significantly improved the outcome, over the control group; → = no significant effect of music therapy was found for the outcome, over the control group; ↓ = the control group significantly improved the outcome, over music therapy; *= the direction of effect is not based on one standardised measure, but a number of subtests where a proportion showed significant results; MI = motor imagery, cMI = metronome-cued motor imagery, CI = confidence interval.

Table S3. Effect direction plot for between-group effects of psychological outcomes for neurological condition (Schünemann et al., 2019) [81].

Studies (Outcome(s) Measured in Study)	Neurological Condition	Cognitive Function (CF)	Mood (M)	Emotion (E)	Behaviour (B)	Quality of Life (QoL)
Raglio et al. (2017) (M, QoL) [11], Segura et al. (2024) (CF, E, M) [33], Poćwierz-Marciniak & Bidzan (2017) (QoL) [57], Chou et al. (2024) (CF, M) [84]	Stroke	→ *	→	↑ *	-	↑ *
Impellizzeri et al. (2024) (CF) [52]	Parkinson’s disease	↑ *	-	-	-	-
Siponkoski et al. (2020) (CF) [53]	Traumatic brain injury	↑ *	-	-	-	-
Van Bruggen-Rufi et al. (2017) (CF, B) [56]	Huntington’s disease	↓	-	-	→	-

Note. Includes 7/10 studies, excluded Haire et al. (2021) [55] due to the lack of comparator group not containing music therapy, and Impellizzeri et al. (2020 [82] and Lee et al. (2024) [54] due to only reliably reporting within-group. ↑ = music therapy significantly improved the outcome, over the control group; → = no significant effect of music therapy was found for the outcome, over the control group; ↓ = the control group significantly improved the outcome, over music therapy; * = the direction of effect is not based on one standardised measure, but a number of subtests where a proportion showed significant results.

Table S4.

Section and Topic	Item #	Checklist Item	Location Where Item is Reported
TITLE			
Title	1	Identify the report as a systematic review.	Title: The Effect of Music Therapy on Psychological Outcomes for Neurological Conditions: Systematic Review (page 1)
ABSTRACT			

Abstract	2	See the PRISMA 2020 for Abstracts checklist.	Abstract done according to PRISMA 2000 guidelines (page 2)
INTRODUCTION			
Rationale	3	Describe the rationale for the review in the context of existing knowledge.	Pages 6–8.
Objectives	4	Provide an explicit statement of the objective(s) or question(s) the review addresses.	Page 8.
METHODS			
Eligibility criteria	5	Specify the inclusion and exclusion criteria for the review and how studies were grouped for the syntheses.	Pages 9–11, Table 2.
Information sources	6	Specify all databases, registers, websites, organisations, reference lists and other sources searched or consulted to identify studies. Specify the date when each source was last searched or consulted.	Pages 9 and 10.; Figure 2
Search strategy	7	Present the full search strategies for all databases, registers and websites, including any filters and limits used.	Pages 10 and 11, Table S1.
Selection process	8	Specify the methods used to decide whether a study met the inclusion criteria of the review, including how many reviewers screened each record and each report retrieved, whether they worked independently, and if applicable, details of automation tools used in the process.	Pages 10 and 11. Figure 2 (PRISMA flow chart)
Data collection process	9	Specify the methods used to collect data from reports, including how many reviewers collected data from each report, whether they worked independently, any processes for obtaining or confirming data from study investigators, and if applicable, details of automation tools used in the process.	Pages 11 and 12,
Data items	10a	List and define all outcomes for which data were sought. Specify whether all results that were compatible with each outcome domain in each study were sought (e.g., for all measures, time points, analyses), and if not, the methods used to decide which results to collect.	Pages 8–10.
	10b	List and define all other variables for which data were sought (e.g., participant and intervention characteristics, funding sources). Describe any assumptions made about any missing or unclear information.	Pages 21–23
Study risk of bias assessment	11	Specify the methods used to assess risk of bias in the included studies, including details of the tool(s) used, how many reviewers assessed each study and whether they worked independently, and if applicable, details of automation tools used in the process.	Page 13, 21–24, 27
Effect measures	12	Specify for each outcome the effect measure(s) (e.g., risk ratio, mean difference) used in the synthesis or presentation of results.	Page 13, Table S2, Table S3

Synthesis methods	13a	Describe the processes used to decide which studies were eligible for each synthesis (e.g., tabulating the study intervention characteristics and comparing against the planned groups for each synthesis (item #5)).	Page 13, and 14. Tables S1–S3, S5 Table 3
	13b	Describe any methods required to prepare the data for presentation or synthesis, such as handling of missing summary statistics, or data conversions.	Page 13 and 14. Tables S2 and S3 Table 3
	13c	Describe any methods used to tabulate or visually display results of individual studies and syntheses.	Page 13 and 14. Tables S2 and S3 Table 3
	13d	Describe any methods used to synthesize results and provide a rationale for the choice(s). If meta-analysis was performed, describe the model(s), method(s) to identify the presence and extent of statistical heterogeneity, and software package(s) used.	Page 13 and 14. Tables S2 and S3
	13e	Describe any methods used to explore possible causes of heterogeneity among study results (e.g., subgroup analysis, meta-regression).	Page 13 and 14. Tables S2 and S3 Table 3
	13f	Describe any sensitivity analyses conducted to assess robustness of the synthesized results.	Page 20, Table S5
Reporting bias assessment	14	Describe any methods used to assess risk of bias due to missing results in a synthesis (arising from reporting biases).	Page 20, Table S5
Certainty assessment	15	Describe any methods used to assess certainty (or confidence) in the body of evidence for an outcome.	Page 13, Table 3
RESULTS			
Study selection	16a	Describe the results of the search and selection process, from the number of records identified in the search to the number of studies included in the review, ideally using a flow diagram.	Pages 14–28 Figure 2
	16b	Cite studies that might appear to meet the inclusion criteria, but which were excluded, and explain why they were excluded.	Page 24
Study characteristics	17	Cite each included study and present its characteristics.	Pages 14–19, including Table 3 (pages 15–19)
Risk of bias in studies	18	Present assessments of risk of bias for each included study.	Pages 20–23
Results of individual studies	19	For all outcomes, present, for each study: (a) summary statistics for each group (where appropriate) and (b) an effect estimate and its precision (e.g., confidence/credible interval), ideally using structured tables or plots.	Table 3 (pages 15–19)
Results of syntheses	20a	For each synthesis, briefly summarise the characteristics and risk of bias among contributing studies.	Pages 20–23

	20b	Present results of all statistical syntheses conducted. If meta-analysis was done, present for each the summary estimate and its precision (e.g., confidence/credible interval) and measures of statistical heterogeneity. If comparing groups, describe the direction of the effect.	N/A—No statistical synthesis due to the SWiM approach
	20c	Present results of all investigations of possible causes of heterogeneity among study results.	Pages 30 and 31
	20d	Present results of all sensitivity analyses conducted to assess the robustness of the synthesized results.	Page 27
Reporting biases	21	Present assessments of risk of bias due to missing results (arising from reporting biases) for each synthesis assessed.	Page 22
Certainty of evidence	22	Present assessments of certainty (or confidence) in the body of evidence for each outcome assessed.	Page 27
DISCUSSION			
Discussion	23a	Provide a general interpretation of the results in the context of other evidence.	Pages 28–31
	23b	Discuss any limitations of the evidence included in the review.	Pages 30 and 31
	23c	Discuss any limitations of the review processes used.	Page 30
	23d	Discuss implications of the results for practice, policy, and future research.	Pages 30 and 31
OTHER INFORMATION			
Registration and protocol	24a	Provide registration information for the review, including register name and registration number, or state that the review was not registered.	Page 31
	24b	Indicate where the review protocol can be accessed, or state that a protocol was not prepared.	Page 31
	24c	Describe and explain any amendments to information provided at registration or in the protocol.	NA (please refer to 24a and 24b)
Support	25	Describe sources of financial or non-financial support for the review, and the role of the funders or sponsors in the review.	Page 31
Competing interests	26	Declare any competing interests of review authors.	Page 31
Availability of data, code and other materials	27	Report which of the following are publicly available and where they can be found: template data collection forms; data extracted from included studies; data used for all analyses; analytic code; any other materials used in the review.	Page 31

From: Page MJ, McKenzie JE, Bossuyt PM, Boutron I, Hoffmann TC, Mulrow CD, et al. The PRISMA 2020 statement: an updated guideline for reporting systematic reviews. BMJ 2021;372:n71. doi: 10.1136/bmj.n71 [72]. This work is licensed under CC BY 4.0. To view a copy of this license, visit https://creativecommons.org/licenses/by/4.0/.(assessed 26 August 2025).

Table S5. Cochrane Risk of Bias (RoB) 2 summary [75].

	**Randomisation Process**	**Deviations from Intended Interventions**	**Missing Outcome Data**	**Measurement of Outcome**	**Selection of the Reported Result**	**Overall Risk of Bias**
Chou et al. (2024) [84]	Low risk (randomised, no group differences at baseline)	Some concerns	Low risk	Some concerns (assessor blinded to participant allocation, self-report measures used)	Low risk	High risk
Haire et al. (2021) [55]	Low risk (randomised, no group differences at baseline)	Low risk	Low risk	Some concerns (assessor blinded to participant allocation, self-report measures used)	Low risk	Some concerns
Impellizzeri et al. (2020) [82]	Low risk (randomised, no group differences at baseline)	Low risk	Low risk	Some concerns (assessor blinded to participant allocation, used self-report measures)	Low risk	Some concerns
Impellizzeri et al. (2024) [52]	Low risk (randomised, differences in Stroop test at baseline, likely due to chance)	Low risk	Low risk	Some concerns (assessor blinded to participant allocation, uses one self-report measure)	Low risk	Some concerns
Lee et al. (2024) [54]	Low risk (randomised, no group differences at baseline)	Low risk	Low risk	Some concerns (assessors not blinded to allocation and familiar with participants, potential for bias)	Low risk	Some concerns
Poćwierz-Marciniak & Bidzan (2017) [57]	Low risk (randomised, no group differences at baseline)	Low risk	Low risk	Some concerns (assessor not blinded to allocation as they delivered the intervention, used self-report measures)	Low risk	Some concerns

Raglio et al. (2017) [11]	Low risk (randomised, no group differences at baseline)	Low risk	Low risk	Some concerns (assessors blinded to participant allocation, used self-report measures)	Low risk	Some concerns
Segura et al. (2024) [33]	Low risk (randomised, no group differences at baseline)	Low risk (intention-to-treat analysis carried out)	Low risk	Some concerns (assessors blinded to participant allocation, used self-report measures)	Low risk	Some concerns
Siponkoski et al. (2020) [53]	Low risk (randomised, only group difference was deviation on cause of injury, but not considered clinically important and attributed to chance)	Low risk (intention-to-treat analysis carried out)	Low risk	Low risk (assessors blinded to participant allocation)	Low risk	Low risk
Van Bruggen-Rufi et al. (2017) [56]	Low risk (randomised, no group differences at baseline)	Low risk (intention-to-treat analysis considered)	Low risk (unclear which groups the participants were allocated to that withdrew due to lack of motivation)	Some concerns (some assessors blinded to participant allocation, not the nursing staff assessing behaviour, observer-reported assessment requiring judgement)	Low risk	Some concerns

Note. Low risk (green) = the study is low risk of bias for all domains. Some concerns (yellow) = the study raises some concerns in at least one domain, but not at high risk for any domain. High risk (red) = the study is at high risk of bias in at least one domain, or the study has some concerns for multiple domains
